# A traveller presenting with severe melioidosis complicated by a pericardial effusion: a case report

**DOI:** 10.1186/1471-2334-12-242

**Published:** 2012-10-04

**Authors:** Detlev Schultze, Brigitt Müller, Thomas Bruderer, Günter Dollenmaier, Julia M Riehm, Katia Boggian

**Affiliations:** 1 Center of Laboratory Medicine, Frohbergstrasse 3, CH-9001 St. Gallen, Switzerland; 2 Department of Internal Medicine, Cantonal Hospital, Rorschacherstrasse 95, CH-9007 St Gallen, Switzerland; 3 Bundeswehr Institute of Microbiology, Neuherbergstr 11, D-80937, Munich, Germany; 4 Department of Internal Medicine, Division of Infectious Diseases, Cantonal Hospital, Rorschacherstrasse 95, CH-9007 St Gallen, Switzerland

**Keywords:** Melioidosis, Burkholderia pseudomallei, Pericardial effusion, Traveller

## Abstract

**Background:**

*Burkholderia pseudomallei*, the etiologic agent of melioidosis, is endemic to tropic regions, mainly in Southeast Asia and northern Australia. Melioidosis occurs only sporadically in travellers returning from disease-endemic areas. Severe clinical disease is seen mostly in patients with alteration of immune status. In particular, pericardial effusion occurs in 1-3% of patients with melioidosis, confined to endemic regions. To our best knowledge, this is the first reported case of melioidosis in a traveller complicated by a hemodynamically significant pericardial effusion without predisposing disease.

**Case presentation:**

A 44-year-old Caucasian man developed pneumonia, with bilateral pleural effusions and complicated by a hemodynamically significant pericardial effusion, soon after his return from Thailand to Switzerland. Cultures from different specimens including blood cultures turned out negative. Diagnosis was only accomplished by isolation of *Burkholderia pseudomallei* from the pericardial aspirate, thus finally enabling the adequate antibiotic treatment.

**Conclusions:**

Melioidosis is a great mimicker and physicians in non-endemic countries should be aware of its varied manifestations. In particular, melioidosis should be considered in differential diagnosis of pericardial effusion in travellers , even without risk factors predisposing to severe disease.

## Background

Melioidosis is a great mimicker and on clinical grounds it is often impossible to differentiate it from other acute and chronic bacterial infections. Definite diagnosis relies on isolation and identification of its causative agent, *Burkholderia pseudomallei*[1,2]. In different endemic regions, pericardial effusion occurs in 1-3% of patients with melioidosis [1]. We present a case of severe melioidosis with a hemodynamically significant pericardial effusion in a traveller returning to a non-endemic region.

## Case presentation

A 44-year-old Caucasian man from Switzerland developed fever and productive cough, two weeks after returning from north-eastern Thailand, were he had stayed from December 2008 until February 2009. The general practitioner treated the patient for community-acquired pneumonia with amoxicillin-clavulanate for seven days. After initial improvement, the patient became febrile and dyspneic again.

On admission the patient was febrile (38.3°C), had a tachycardia of 130 beats/minute, a blood pressure of 120/78 mmHg, and a respiratory rate of 40/min.

Although the patient showed jugular venous distention, neither Kussmaul’s sign nor hepatomegaly or peripheral oedema were observed.

Laboratory tests revealed anaemia (hemoglobin 125 g/l, hematocrit 0.37), leucocytosis, (16.6 G/l; 80% neutrophils, 12% lymphocytes), elevated C-reactive protein (141 mg/l) and elevated B-type natriuretic peptide (208 ng/l). Laboratory screening for autoimmune diseases and vasculitis was negative. Electrocardiogram showed sinus tachycardia and low QRS voltage.

A chest radiograph showed bilateral pleural effusions and an enlarged cardiac silhouette. Computed tomography (CT) of the chest confirmed bilateral pleural effusions, with atelectasis of the inferior lobes, mediastinal lymphadenopathy and a prominent pericardial effusion. Abdominal CT showed a small intra-abdominal fluid collection.

Echocardiography confirmed a hemodynamic relevant pericardial effusion with diastolic compression of the right ventricle and a leftventricular ejection fraction of 55%. After pericardiocentesis and aspiration of 700 ml of a clear yellowish fluid the right ventricular function normalized, the leftventricular ejection fraction raised to 65%, and the QRS voltage normalized.

Pleural effusion (1.07 G/l leucocytes, 33% monocytes/macrophages, 54% lymphocytes, 13% polymorphonuclear neutrophil leucocytes; LDH 144 U/l with normal range of LDH in serum <265 U/l ,and with a pleural fluid/serum-quotient of 0.4 for LDH and 0.4 for total protein, respectively) was negative on Gram- and Ziehl-Neelsen stains and negative by Polymerase Chain Reaction Assay for *Mycobacterium tuberculosis* complex. Cultures remained negative for bacteria, including mycobacteria and fungi. Cultures of two sputum samples from the same day yielded normal upper respiratory tract flora. Four sets of blood cultures taken on four consecutive days remained sterile and urine was negative for Legionella antigen.

Pericardial effusion (1.4 G/l leucocytes, 58% monocytes/macrophages, 23% lymphocytes, 19% polymorphonuclear neutrophil leucocytes; pericardial fluid/serum-quotient of 0.6 for total protein content and of 2.3 for LDH activity, respectively) was negative on Ziehl-Neelsen stain and mycobacterial cultures remained negative. Two blood culture sets were inoculated with pericardial aspirate (10 ml volume per bottle), and a Gram-negative bacillus was isolated from both aerobic bottles after 35 hours of incubation in a BACTEC™ http://9240 Blood Culture Analyzer (Becton Dickinson AG, Allschwil, Switzerland). Although identified as *Burkholderia cepacia* in a UNMIC/ID-62 panel of the Phoenix Automated Microbiology System (Becton Dickinson AG, Allschwil, Switzerland), diagnosis was regarded as tentative, since identification of *B. cepacia* by common automated identification instruments should be confirmed by molecular tests [3,4]. Furthermore, pericardial effusion is an unusual location for occurrence of *B. cepacia*[3] and the isolate was unexpectedly sensitive to amoxicillin-clavulanate (MIC <= 4/2 mg/L). Analysis of the isolate by API 20NE biochemical test panel V7.0 (bioMérieux, Geneva, Switzerland) yielded *Burkholderia pseudomallei* (profile 1156577; 99.9%, ID, 1.0 T). Amplification and sequencing of a 500-bp fragment of the 16S rRNA gene by Fast MicroSeq 500 16S rDNA Bacterial Identification Kit and a PRISM 3130 Genetic Analyzer (Applied Biosystems, Foster City, CA, USA) with sequence analysis by MicroSeq ID Microbial Identification Software (Applied Biosystems, Foster City, CA, USA) confirmed the isolate as *B. pseudomallei* (DQ108392, 481-bp consensus length). Multilocus sequence typing (MLST) [5] of the isolate revealed the allelic profile 1/1/4/1/5/4/1, corresponding to *B. pseudomallei* sequence type 207, which has first been isolated from a patient in Thailand with invasive melioidosis in 2001 [6]. The isolate was sensitive to amoxicillin-clavulanate (2 μg/mL), ceftazidime (1.5 μg/mL), doxycycline (3 μg/mL) and trimethoprim-sulfamethoxazole (0.75/0.0375 μg/mL). Susceptibility testing was carried out by Etest (AB BIODISK, Sweden) and interpreted according to guidelines established by CLSI [7].

The patient received ceftazidime 2 g every 6 hours for 2 weeks followed by maintenance treatment for three months with doxycycline, trimethoprim-sulfamethoxazole and leucovorine. The patient fully recovered after four months and suffered no relapse in the two years follow-up.

## Discussion

Our patient developed signs of respiratory illness within the usual incubation period for melioidosis of 1-21 days, shortly after leaving northeastern Thailand, an endemic region for this illness. As Switzerland is not among those countries, where autochthonous cases of melioidosis have been reported [1], the patient probably acquired the infection during his stay in Thailand.

Pneumonia results from haematogenous spread of *B. pseudomallei* to the lung following inoculation through exposure to muddy soils or surface water or alternatively by inhalation, as the two main modes of infection [1,8].

Although melioidosis occurs in all age groups, severe clinical disease, such as septicaemic pneumonia, is seen mostly in patients with alteration of immune status, e.g. diabetes, chronic renal failure or alcoholism [8]. Our patient had neither a clinical risk factor for melioidosis nor any other underlying disease.

Epidemiologic considerations are very important in the management of pericardial effusion, as in developed countries acute idiopathic pericarditis and idiopathic pericardial effusion are the most common etiologies, whereas in some underdeveloped geographic areas tuberculous pericarditis is the leading cause of pericardial effusion [9]. In a systematic analysis of 106 pericardial fluid samples from France, a non-endemic country for melioidosis, *B. pseudomallei* was not among the detected bacterial agents [10]. Even in endemic regions, pericardial effusion caused by *B. pseudomallei* is a rare phenomenon. In the Darwin prospective melioidosis study from tropical Australia only four of 540 documented cases had pericarditis, three of them with pulmonary infection [11]. In a 10-years retrospective study from Thailand only 12 domestic cases of melioidosis complicated by culture-confirmed pericarditis were found. One-third of these patients had underlying diseases and two-third showed evidence of bacteremia with secondary seeding in the pericardium [12]. In areas where tuberculosis and melioidosis are endemic, complicating pericarditis may only be differentiated by pericardial fluid culture and findings of pericardial biopsy [12].

As bacteremia could not be detected in our patient, *B. pseudomallei* might have gained access into the pericardium through the mediastinal lymph nodes in the course of pneumonia, known from patients with tuberculosis [13]. Cultures from different specimens remained negative for *B. pseudomallei*. Only culture of pericardial fluid grew the causative agent, possibly due to the use of blood culture bottles as primary culture medium. Blood culture bottles are superior in performance to traditional plated-medium methods for detection of microorganisms from sterile body fluids [14]. Detection of *B. pseudomallei* from nonsterile specimens such as sputum samples, can be hampered by the overgrowth and masking of *B. pseudomallei* by commensal flora, if selective culture media are not used, that are more common in medical laboratories situated in countries where the disease is endemic [1].

The initial empiric therapy with orally given amoxicillin-clavulanate was certainly inadequate as an intensive phase therapy for melioidosis [1,2]. However, this may have been responsible for the absence of growth of *B. pseudomallei* in other samples, especially in blood culture. This may also explain why the patient did not develop a septic shock as recently reported to be very common in patients with primary pneumonia combined with positive blood culture [15]. The detection of *B. pseudomallei* in the pericardial aspirate finally enabled the adequate, although delayed antibiotic treatment, 22 days after onset of illness.

## Conclusions

Melioidosis is a great mimicker and physicians in non-endemic countries like Switzerland should be aware of its varied manifestations. In particular, melioidosis should be considered in the differential diagnosis of pericardial effusion in travellers, even in the absence of risk factors predisposing to severe disease. To our best knowledge, this is the first reported case of melioidosis in a traveller complicated by a hemodynamically significant pericardial effusion.

## Consent

Written informed consent was obtained from the patient for publication of this case report. A copy of the written consent is available for review by the Editor-in-Chief of this Journal.

## Competing interests

The authors declare that they have no competing interests.

## Authors’ contributions

DS supervised the microbiological analyses and wrote the manuscript. ThB, GD, JMR supervised the microbiological analyses and helped to draft the manuscript. BM and KB contributed to diagnosis and treatment and KB helped to draft the manuscript and did outpatient follow-up. All authors read and approved the final manuscript.

## Pre-publication history

The pre-publication history for this paper can be accessed here:

http://www.biomedcentral.com/1471-2334/12/242/prepub
